# Comparative outcomes of natural orifice specimen extraction surgery versus totally laparoscopic surgery for right-sided colon cancer: a single-centre propensity score-matched study

**DOI:** 10.3389/fsurg.2026.1716425

**Published:** 2026-02-25

**Authors:** Zheng Xu, Yueyang Zhang, Jian Ma, Changyuan Gao, Haipeng Chen, Jianwei Liang, Zhaoxu Zheng, Xu Guan, Haitao Zhou, Xishan Wang

**Affiliations:** Department of Colorectal Surgery, National Cancer Center/National Clinical Research Center for Cancer/Cancer Hospital, Chinese Academy of Medical Sciences and Peking Union Medical College, Beijing, China

**Keywords:** colon cancer, long-term prognosis, natural orifice specimen extraction surgery, postoperative complications, right hemicolectomy

## Abstract

**Aim:**

To evaluate the safety, feasibility, and long-term efficacy of natural orifice specimen extraction surgery (NOSES) compared with totally laparoscopic right hemicolectomy (TLRH) for right-sided colon cancer.

**Methods:**

This single-center retrospective study included 349 patients who underwent laparoscopic curative resection for stage I-III right-sided colon cancer between January 2018 and January 2023. After 1:1 propensity score matching (PSM) for age, tumor size, BMI, neoadjuvant therapy, and T stage, 115 NOSES patients were compared with 115 TLRH patients. Outcomes included postoperative recovery, perioperative fatigue, complications, pelvic floor function, disease-free survival (DFS), and overall survival (OS).

**Results:**

After PSM, baseline characteristics were balanced. Operative time and blood loss did not differ between groups. NOSES was associated with significantly less postoperative pain (*P* < 0.001) and lower analgesic use (25.2% vs. 47.0%, *P* < 0.001). Learning curves indicated proficiency after 57 transvaginal and 32 transrectal procedures. Recovery indicators, including time to first flatus, defecation, and hospital stay, were comparable. Incision-related complications occurred more frequently in TLRH (*P* = 0.024). NOSES patients reported lower fatigue levels on postoperative days 1 and 3 (*P* < 0.001), with fewer cases of postoperative fatigue syndrome. Pelvic floor and continence outcomes were similar. No local recurrences were observed, and DFS and OS did not differ significantly.

**Conclusions:**

NOSES is a safe and effective alternative for selected patients with right-sided colon cancer. It reduces postoperative pain, fatigue, and incision-related complications without compromising oncological outcomes or pelvic floor function, and demonstrates a clear learning curve supporting its broader application.

## 1 Introduction

Colorectal cancer remains a major global health challenge. In 2022, an estimated 1.9 million new cases and 904,000 deaths were reported (1), making it the third most common cancer worldwide and the second leading cause of cancer-related mortality. Advances in surgical techniques have significantly improved patient outcomes in recent years. Among these, laparoscopic-assisted radical colectomy has become the standard approach for colon cancer surgery due to its proven safety and efficacy (2). In conventional laparoscopic surgery, anastomosis and mesenteric division are performed extracorporeally, thereby necessitating a larger abdominal incision. By contrast, totally laparoscopic right hemicolectomy (TLRH), which involves performing intracorporeal anastomosis and specimen extraction through a small abdominal incision, further enhances the benefits of minimally invasive surgery (3, 4). These include reduced postoperative pain, faster recovery, and fewer complications compared to traditional open surgery, while also addressing limitations of laparoscopic-assisted techniques (5).

The growing interest in minimally invasive approaches has also led to the emergence of natural orifice specimen extraction surgery (NOSES) as a promising alternative (6). By enabling specimen retrieval through natural orifices, such as the vagina, NOSES eliminates the need for large abdominal incisions. A retrospective study of 5,055 colorectal tumor cases demonstrated the safety, feasibility, and favorable short-term oncological outcomes of this technique (7). Beyond oncological safety, NOSES offers several postoperative advantages, including faster recovery of bowel function, reduced pain, improved cosmetic outcomes, and enhanced psychosocial well-being (8, 9). Moreover, certain variations of NOSES have also demonstrated safety and effectiveness in selected patient populations (10). While these benefits are well-documented in rectal cancer surgeries, the application and effectiveness of NOSES in right-sided colon cancer—especially in comparison to TLRH—remain uncertain.

Despite the established advantages of both TLRH and NOSES in reducing surgical trauma, their comparative efficacy in managing right-sided colon cancer remains underexplored. While TLRH has demonstrated strong outcomes, NOSES may further enhance postoperative recovery by completely avoiding abdominal incisions, potentially surpassing TLRH in minimizing pain and recovery time (11). Efetov and his colleagues reported that combining reduced-port laparoscopic surgery with NOSES resulted in faster postoperative recovery and may be suitable for patients with right-sided colon cancer (12). However, most current research on NOSES is limited to rectal cancer, with studies often constrained by small sample sizes and short follow-up periods (13). This creates a significant gap in evidence regarding its long-term outcomes and specific benefits for right colon resections.

A recent meta-analysis by Pompeu et al. demonstrated that, compared with laparoscopic right hemicolectomy, NOSES is associated with reduced postoperative pain, lower surgical site infection rates, and faster bowel recovery (14). In parallel, Zhang et al. reported favorable perioperative outcomes for transrectal NOSES compared with laparoscopic colectomy (15). Despite these encouraging results, most existing studies have focused on comparisons with laparoscopic techniques or extracorporeal anastomosis, and data directly comparing NOSES with totally laparoscopic right hemicolectomy with intracorporeal anastomosis remain limited. Building on prior evidence, the present study applies PSM to compare NOSES and TLRH, with a comprehensive evaluation of both short-term perioperative outcomes and long-term oncologic and functional results. Additionally, we incorporated fatigue and functional recovery measures and performed a quantitative assessment of the NOSES learning curve. This study thus addresses a critical gap by systematically evaluating the strengths of NOSES in right-sided colon cancer surgeries, clarifying its role relative to TLRH, and highlighting its potential impact on both recovery and surgical outcomes over the long term.

## 2 Method

### 2.1 Study design and patient selection

We reviewed prospectively collected data from all patients who underwent laparoscopic curative resection for stage I–III right-sided colon cancer between January 2018 and January 2023 at Cancer Hospital, Chinese Academy of Medical Sciences and Peking Union Medical College. A total of consecutive 115 patients who underwent laparoscopic resection with transvaginal or transrectal specimen extraction were identified as the NOSES group. To establish a comparative cohort, 234 patients who underwent TLRH during the same period were selected. Both NOSES and TLRH procedures were performed by the same surgeon and surgical team throughout the study period. Propensity scores, calculated based on age, tumor size, body mass index (BMI), neoadjuvant therapy, and T stage (American Joint Committee on Cancer, 8th edition), were used to perform 1:1 matching. This resulted in 115 matched patients in the TLRH group, forming a balanced cohort for comparison. Comprehensive medical histories were recorded for all patients, and preoperative evaluations, including chest, abdominal, and pelvic CT scans as well as colonoscopy, were performed to confirm clinical staging and surgical eligibility.

Eligibility for NOSES was determined based on international consensus guidelines for natural orifice specimen extraction surgery (NOSES) in colorectal cancer (16). Patients were included if they met the following criteria: i) Imaging-confirmed T1–3 colon cancer with a tumor diameter ≤5 cm; ii) No active vaginal infection, malformation, or stenosis; iii) History of childbirth with no further fertility requirements. Exclusion criteria included locally advanced cancer, tumor size exceeding 5 cm, or BMI >30 kg/m^2^. Eligible patients were informed of the potential benefits and risks of NOSES and TLRH and provided informed consent if they opted for the NOSES approach. The final decision to proceed with NOSES was made intraoperatively based on surgical feasibility, and only successfully completed NOSES cases were included in the analysis. The study was registered on ClinicalTrials.gov (NCT06753968) and received the ethical approval from the Ethics Committee of the National Cancer Center/Cancer Hospital, Chinese Academy of Medical Sciences and Peking Union Medical College (approval number: NCC2022072).

### 2.2 Data collection

The main indicators included patient demographics, disease-related features, pathological characteristics, surgical details, and short-term outcomes. Demographic and disease-related factors, such as age, gender, body mass index (BMI), tumor location, comorbidities, history of abdominal surgery, preoperative neoadjuvant therapy, and carcinoembryonic antigen (CEA) levels, were initially assessed. Pathological characteristics, including histological type, differentiation grade, tumor-node-metastasis (TNM) stage (according to the American Joint Committee on Cancer), tumor size, and the number of harvested and positive lymph nodes, were then examined. Surgical characteristics, such as the duration of surgery, estimated blood loss, and length of the skin incision, were also analyzed. Finally, short-term (30-day) outcomes were evaluated, focusing on postoperative complications, pain scores (measured using the Visual Analog Scale, VAS), fatigue levels, time to first flatus, time to first defecation, postoperative length of stay, and rates of reoperation and readmission.

### 2.3 Surgical procedures

In both the TLRH and NOSES groups, the surgical approach and steps prior to specimen removal were identical, with the standard procedure involving a medial-to-lateral approach for complete mesocolic excision, performed by experienced surgeons. Specifically, after general anesthesia, for the intracorporeal anastomosis, after transecting the transverse colon and terminal ileum with endoscopic linear staplers, two small incisions (10 mm) were made along the curvature of the intestinal ends on the anti-mesenteric side. The intestinal ends were then approximated and connected using a 60-mm linear stapler, and the common enterotomy was sealed with another linear stapler.

In the TLRH group, following the stapled side-to-side anastomosis, a small horizontal incision is made about 2–3 cm above the symphysis pubis, at the junction of the pubic hairline, to allow for specimen removal. In contrast, the NOSES group employs two distinct methods for specimen extraction, both of which have been thoroughly outlined in previous studies (15, 17). The first method, transvaginal extraction, begins with the irrigation and disinfection of the vagina, followed by a transverse incision in the posterior fornix. The assistant then utilizes oval forceps to carefully extract the specimen bag through the incision, which is subsequently closed with continuous full-thickness sutures, using barbed sutures to ensure secure closure. The second method, transrectal extraction, similarly begins with irrigation of the rectum using a dilute iodine solution, followed by a longitudinal incision along the anterior wall of the upper rectum. The specimen, along with the protective sleeve, is then removed using oval forceps, and the incision is closed with a continuous full-layer running suture once the specimen has been completely extracted. To prevent tumor spillage during specimen extraction, all NOSES procedures were performed using a protective extraction bag and the no-touch technique.

After specimen extraction, the abdominal cavity was thoroughly irrigated with 500 mL of normal saline to minimize the risk of contamination. Routine bacteriological sampling was not performed unless intraoperative contamination was suspected (16). The incision area was then immediately covered with standard sterile dressings, which were replaced every 1–3 days depending on the amount of exudate.

### 2.4 Perioperative management and follow-up

In this study, neoadjuvant therapy was administered exclusively to patients with locally advanced colon cancer (M0), defined as T3–T4 with extramural invasion ≥5 mm. The treatment regimen comprised oxaliplatin at 130 mg/m^2^ delivered as a 1-hour intravenous infusion on day 1, combined with oral capecitabine at 1,000 mg/m^2^ twice daily on days 1–14. The cycle was repeated every 3 weeks for a total of two courses prior to surgery (18, 19). Perioperative management was consistent across all cases. On postoperative Days 1 and 2, patient-controlled analgesia was provided, supplemented by non-steroidal anti-inflammatory drugs as additional pain relief. Bowel recovery was monitored through the passage of flatus and stool, and once motility was restored, patients were allowed to resume eating. Pain levels were recorded on Days 1, 3, and 5 after surgery using the VAS (20). To assess postoperative fatigue, the Christensen Fatigue Scale was employed for both the TLRH and NOSES groups (21). A score of 6 or higher was considered indicative of fatigue (22). Evaluations were conducted at four intervals: one day prior to surgery, and on the first, third, and seventh days following the operation. Postoperative complications, occurring up to the one-month follow-up, were documented and classified according to the Clavien-Dindo grading system. Pelvic floor function was measured preoperatively and at 3-month and 1-year postoperative follow-up using the Pelvic Floor Distress Inventory Short Form 20 (PFDI-20), which includes the Pelvic Organ Prolapse Distress Inventory 6 (POPDI-6), Colorectal-Anal Distress Inventory 8 (CRADI-8), and Urinary Distress Inventory 6 (UDI-6). These instruments assessed the extent of symptom impact on quality of life, with higher scores reflecting greater impairment (23). Additionally, the severity of fecal incontinence was evaluated with the Wexner score, which assesses the frequency and type of incontinence (solid, liquid, and flatus) as well as its effect on daily living, such as the need to wear a pad and lifestyle alteration (24). Follow-up visits were scheduled in accordance with National Comprehensive Cancer Network guidelines: one month after surgery, every 3 months for the first 2 years, and every 6 months for the subsequent 5 years. Long-term outcomes were assessed in terms of disease-free survival (DFS), defined as the duration from surgery to relapse, metastasis, or death from any cause, and overall survival (OS).

### 2.5 Learning curve analysis of NOSES

A learning curve analysis of NOSES procedures was conducted using perioperative data from a surgical team performing over 20 NOSES procedures annually for colorectal cancer patients. The team consisted of a senior surgeon with 30 years of oncology and laparoscopic colorectal surgery experience, supported by two consistent assistants throughout the study.

Operation time (OT) was used as the primary metric to assess surgical skill development and performance. To analyze the relationship between the surgeon's NOSES experience and OT, the cumulative sum (CUSUM) analyses were conducted. In the CUSUM analysis, cases were ordered chronologically, with transvaginal and transrectal procedures assessed separately. The initial CUSUM_OT_ was calculated as the difference between the OT of the first case and the overall mean OT. For subsequent cases, CUSUM_OT_ was derived by adding the deviation of each OT from the mean to the cumulative total from prior cases (25). This iterative calculation produced a graphical representation of deviations from the mean OT, with inflection points—defined by four or more consecutive negative values—marking transitions in the learning curve and dividing it into distinct phases, offering a comprehensive evaluation of the surgeon's learning curve and progression in NOSES procedures.

### 2.6 Statistical analysis

Continuous variables that followed a normal distribution were presented as mean ± standard deviation and analyzed using Student's *t*-test, while non-normally distributed continuous variables were reported as medians with interquartile range. Categorical variables were expressed as percentages and analyzed using either Fisher's exact test or the chi-square test, depending on the nature of the data. Given that NOSES can only be applied to selected patients, PSM was used to ensure appropriate patient comparability between groups. PSM was performed using 1:1 optimal matching without replacement, based on a multivariable logistic regression model. A caliper width of 0.2 standard deviations of the logit of the propensity score was applied to minimize poor matches. After matching, covariate balance was assessed using standardized mean differences (SMDs), with all included covariates achieving values below 0.1, indicating adequate balance. Propensity scores were estimated using multivariable logistic regression incorporating clinically relevant variables selected *a priori* based on prior studies (17, 26). These covariates included age, BMI, tumor size, T stage, and receipt of neoadjuvant therapy. Kaplan–Meier curves were used to assess long-term outcomes. A *P*-value of <0.05 was considered statistically significant. All statistical analyses were performed using R software (version 4.0.4; R Foundation for Statistical Computing, Vienna, Austria, https://www.R-project.org) for PSM, and SPSS software (version 25.0.0; SPSS Inc., Chicago, IL, USA) for the remaining analyses.

## 3 Results

### 3.1 Baseline characteristics

Prior to PSM, a total of 349 patients were included in the analysis, with 234 in the TLRH group and 115 in the NOSES group (Table 1). Several baseline characteristics exhibited significant imbalances between the two groups. Patients in the NOSES group had a significantly lower BMI compared to those in the TLRH group (23.2 ± 3.4 vs. 23.9 ± 2.7 kg/m², *P* = 0.013). The proportion of patients who received preoperative therapy was also significantly lower in the NOSES group (4.3% vs. 11.1%, *P* = 0.037). Additionally, tumor size (*P* = 0.002) and T stage distribution (*P* = 0.030) differed significantly, with the NOSES group having a lower proportion of T4 tumors.

**Table 1:** Baseline data of patients in the TLRH and NOSES group.

Variable	Total	Before matching		*P*	After PSM		*P*
		TLRH	NOSES		TLRH	NOSES	
	(*n* = 349)	(*n* = 234)	(*n* = 115)		(*n* = 115)	(*n* = 115)	
Sex, *n* (%)				0.167			0.185
Female	188 (53.9)	120 (51.3)	68 (59.1)		57 (49.6)	68 (59.1)	
Male	161 (46.1)	114 (48.7)	47 (40.9)		58 (50.4)	47 (40.9)	
Age, yr	61 (53–68)	63 (56–70)	60 (52–68)	0.705	63 (56–71)	60 (52–68)	0.266
BMI, kg/m^2^	23.9 ± 3.2	23.9 ± 2.7	23.2 ± 3.4	**0** **.** **013**	23.6 ± 2.8	23.2 ± 3.4	0.120
Preoperative CEA, ng/mL	2.8 (0.1–5.6)	2.9 (0.4–5.5)	2.9 (0.4–5.5)	0.878	2.9 (0.3–5.5)	2.9 (0.1–5.8)	0.803
Comorbidity, *n* (%)				0.393			0.563
No	250 (71.6)	171 (73.1)	79 (68.7)		83 (72.2)	79 (68.7)	
Yes	99 (28.4)	63 (26.9)	36 (31.3)		32 (27.8)	36 (31.3)	
Preoperative therapy, *n* (%)				**0** **.** **037**			0.553
No	318 (92.8)	208 (88.9)	110 (95.7)		108 (93.9)	110 (95.7)	
Yes	31 (7.2)	26 (11.1)	5 (4.3)		7 (6.1)	5 (4.3)	
Abdominal history, *n* (%)				0.650			0.624
No	278 (79.7)	188 (80.3)	90 (78.3)		93 (80.9)	90 (78.3)	
Yes	50 (20.3)	46 (19.7)	25 (21.7)		22 (19.1)	25 (21.7)	
Tumor histology, *n* (%)				0.341			0.710
Adenocarcinoma	291 (83.4)	192 (82.1)	99 (86.1)		97 (84.3)	99 (86.1)	
Mucinous tumor	58 (16.6)	42 (17.9)	16 (13.9)		18 (16.7)	16 (13.9)	
Tumor differentiation, *n* (%)				**0** **.** **063**			0.124
Good	17 (4.9)	8 (3.4)	9 (7.8)		4 (3.5)	9 (7.8)	
Moderate	177 (50.7)	114 (48.7)	63 (54.8)		55 (47.8)	63 (54.8)	
Poor	155 (44.4)	112 (47.9)	43 (37.4)		56 (48.7)	43 (37.4)	
Tumor size, cm	4 (3–5)	4 (3–5)	4 (3–5)	**0** **.** **002**	4 (3–5)	4 (3–5)	0.331
Tumor location, *n* (%)				0.823			0.416
Ileocecum	81 (23.2)	53 (22.6)	28 (24.3)		31 (27.0)	28 (24.3)	
Ascending colon	217 (62.2)	145 (70.0)	72 (62.6)		63 (54.8)	72 (62.6)	
Hepatic flexure	51 (14.6)	36 (15.4)	15 (13.1)		21 (18.3)	15 (13.1)	
T stage, *n* (%)				**0**.**030**			0.251
T1	46 (13.2)	32 (13.7)	16 (13.9)		15 (13.0）	16 (13.9)	
T2	46 (13.2)	24 (10.3)	20 (17.4)		13 (11.3)	20 (17.4)	
T3	139 (39.8)	90 (38.5)	49 (42.6)		44 (38.3)	49 (42.6)	
T4	118 (33.8)	88 (37.6)	30 (26.1)		43 (37.4)	30 (26.1)	
N stage, *n* (%)				0.278			0.381
N0	218	145 (62.0)	73 (63.5)		73 (63.5)	73 (63.5)	
N1	84	53 (22.6)	31 (27.0)		25 (21.7)	31 (27.0)	
N2	47	36 (15.4)	11 (9.5)		17 (14.8)	11 (9.5)	
AJCC TNM stage, *n* (%)				0.610			0.588
I	78 (22.3)	50 (21.4)	28 (24.3)		23 (20.0)	28 (24.3)	
II	134 (38.4)	90 (38.5)	43 (37.4)		50 (43.5)	43 (37.4)	
III	137 (39.2)	93 (39.7)	44 (38.3)		42 (36.5)	44 (38.3)	
Nerve invasion, *n* (%)				0.401			0.148
No	241 (69.1)	165 (70.5)	76 (66.1)		86 (74.8)	76 (66.1)	
Yes	108 (30.9)	69 (29.5)	39 (33.9)		29 (25.2)	39 (33.9)	
Vascular invasion, *n* (%)				0.431			0.450
No	261 (74.8)	178 (76.1)	83 (72.2)		88 (76.5)	83 (72.2)	
Yes	88 (25.2)	56 (23.9)	32 (27.8)		27 (23.5)	32 (27.8)	
Extramural vascular invasion, *n* (%)				0.412			0.582
No	299(85.7)	203(86.8)	96(83.5)		99(86.1)	96(83.5)	
Yes	50(14.3)	31(13.2)	19(16.5)		16(13.9)	19(16.5)	

Values are presented as mean ± SD, median and interquartile range, or numbers (%).

PSM, propensity score matching; BMI, body mass index; CEA, carcinoembryonic antigen; AJCC, American Joint Committee on Cancer.

Bold values indicate *P* < 0.05.

After performing 1:1 PSM, a total of 115 well-matched patient pairs were included in the final analysis. Following PSM, sex distribution was similar between groups (*P* = 0.185). The previously significant difference in BMI was no longer present, with mean values of 23.6 ± 2.8 kg/m^2^ in the TLRH group and 23.2 ± 3.4 g/m^2^ in the NOSES group (*P* = 0.120). The imbalance in the proportion of patients receiving preoperative therapy was also resolved, with rates of 6.1% in the TLRH group and 4.3% in the NOSES group (*P* = 0.553). Tumor size differences that were significant before matching were no longer statistically relevant (median: 4 cm in both groups, *P* = 0.331). Similarly, T stage distribution, which was significantly different before matching, became well-balanced after PSM (*P* = 0.251). Beyond these key variables, other baseline characteristics—including age, preoperative CEA levels, comorbidities, history of abdominal surgery, tumor histology, tumor location, N stage, AJCC TNM stage, nerve invasion, vascular invasion, and extramural vascular invasion—showed no statistically significant differences between the two groups after PSM.

### 3.2 Perioperative and early postoperative outcomes

The perioperative and early postoperative outcomes of patients undergoing TLRH and NOSES were compared (Table 2). Overall, NOSES demonstrated clear advantages in postoperative pain control and incision-related complications, while operative parameters, recovery profiles, and oncologic surrogates were comparable between groups.

**Table 2:** Intraoperative and postoperative conditions in patients with TLRH and NOSES.

Variable	TLRH	NOSES	*P*
	(*n* = 115)	(*n* = 115)	
Operative time, min	151 (123–180)	161 (129–193)	0.191
Estimated blood loss, mL	25 (18–33)	25 (15–35)	0.382
Length of resected ileum, cm	10 (6–14)	12 (9–15)	0.130
Length of resected colon, cm	22 (19–25)	22 (17–28)	0.535
Harvested lymph nodes, *n* (%)	34 (26–47)	32 (18–47)	0.527
Positive lymph nodes, *n* (%)	0 (0–1)	0 (0–1)	0.698
Usage of additional analgesics, *n* (%)	54 (47.0)	29 (25.2)	**<0** **.** **001**
VAS score			
POD 1	4 (2–6)	2 (1–4)	**<0** **.** **001**
POD 2	2 (1–4)	2 (0–3)	**0** **.** **021**
POD 3	2 (1–3)	1 (0–2)	**<0** **.** **001**
1st flatus, d	2 (2–3)	2 (2–3)	0.785
1st defecation, d	4 (4–5)	4 (4–5)	0.880
Postoperative hospital stay, d	6 (4–7)	6 (5–7)	0.754
Postoperative complication, *n* (%)			0.858
Grade 1	12 (10.4)	13 (11.3)	
Grade 2	3 (2.6)	2 (2.6)	
Grade 3	3 (3.6)	3 (3.6)	
Grade 4	1 (0.9)	1 (0.9)	
Anastomotic leak, *n* (%)	1 (0.9)	1 (0.9)	1.000
Anastomotic bleeding, *n* (%)	2 (1.7)	3 (2.6)	0.651
Intestinal obstruction, *n* (%)	2 (1.7)	2 (1.7)	1.000
Abdominal infection, *n* (%)	1 (0.9)	2 (2.6)	0.561
Incision-related infections, *n* (%)	5 (4.3)	0 (0)	**0** **.** **024**
Admitted to ICU, *n* (%)	2 (1.7)	2 (1.7)	1.000
Reoperation, *n* (%)	1 (0.9)	0 (0)	0.316
Readmission, *n* (%)	1 (0.9)	0 (0)	0.316

Values are presented as median and interquartile range, or numbers (%).

VAS, visual analog scale; POD, postoperative day.

Bold values indicate *P* < 0.05.

The median operative time was slightly longer in the NOSES group compared to the TLRH group [161 (129–193) min vs. 151 (123–180) min, *P* = 0.191], though the difference was not statistically significant. Estimated intraoperative blood loss was similar between groups [25 (15–35) mL vs. 25 (18–33) mL, *P* = 0.382]. The lengths of resected ileum and colon were comparable, with no significant differences observed. Similarly, the number of harvested lymph nodes and positive lymph nodes did not differ significantly between groups (*P* = 0.527 and *P* = 0.698, respectively).

Postoperative pain, as measured by the VAS, was significantly lower in the NOSES group. On postoperative day (POD) 1, the median VAS score was 2 (1–4) in the NOSES group compared to 4 (2–6) in the TLRH group (*P* < 0.001). This trend continued on POD 2 and POD 3, with lower pain scores in the NOSES group (*P* = 0.021 and *P* < 0.001, respectively). Additionally, the use of additional analgesics was significantly lower in the NOSES group (25.2% vs. 47.0%, *P* < 0.001).

Postoperative recovery was assessed using indicators such as time to first flatus, first defecation, and length of hospital stay. The time to first flatus (*P* = 0.785), first defecation (*P* = 0.880) and postoperative hospital stay (*P* = 0.754) were similar. Regarding postoperative complications, there were no significant differences between the two groups in overall complication rates (*P* = 0.858). The incidence of major complications, including anastomotic leak (*P* = 1.000), anastomotic bleeding *(P* = 0.651), intestinal obstruction (*P* = 1.000), abdominal infection (*P* = 0.561), admission to the ICU (*P* = 1.000), reoperation (*P* = 0.316), and readmission (*P* = 0.316), was comparable between groups. Notably, incision-related infection occurred more frequently in the TLRH group [5 (4.3%) vs. 0 (0%), *P* = 0.024], while no rectal or vaginal complications were observed in the NOSES group.

### 3.3 Learning curve analysis

**Figure 1:**
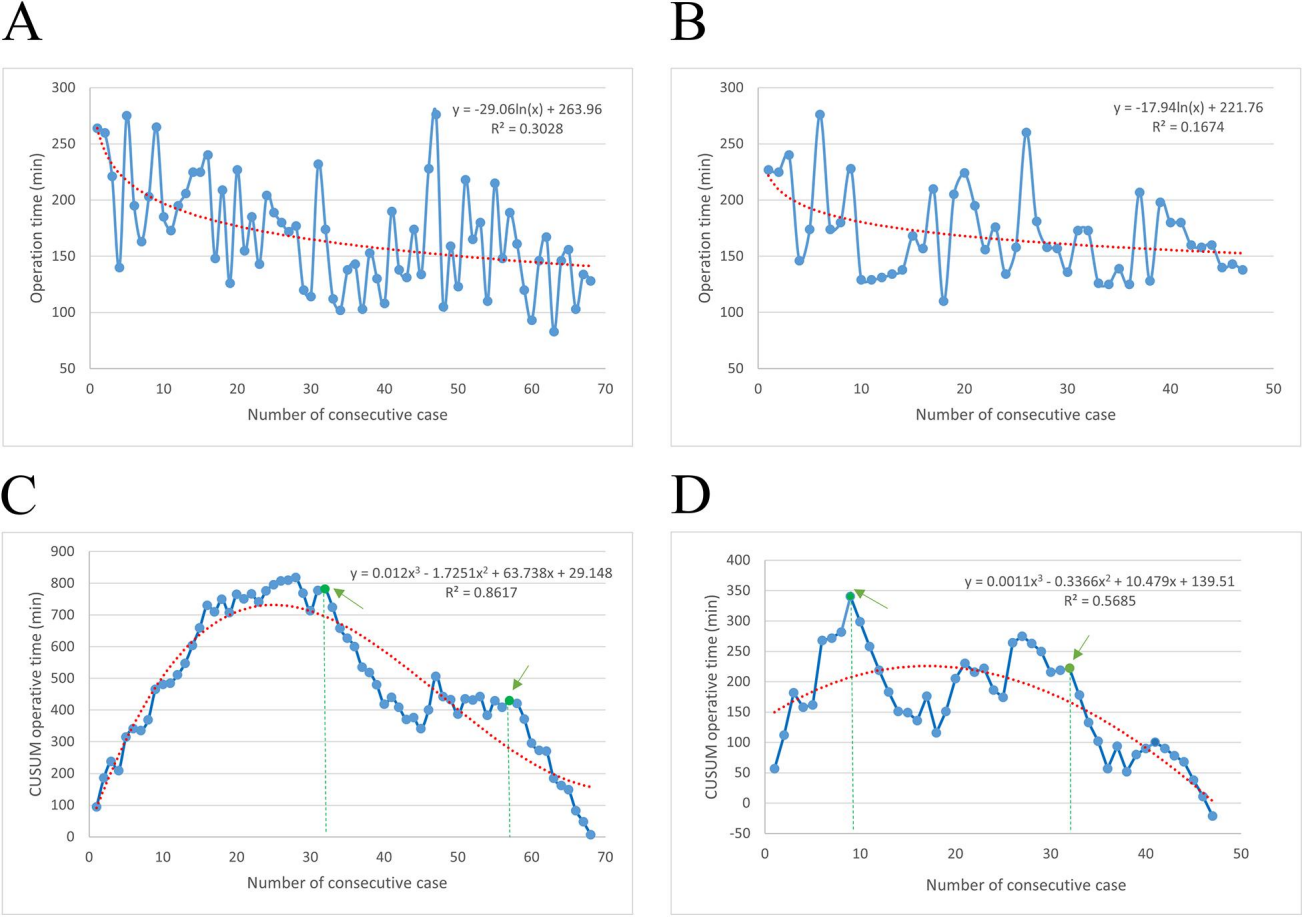
Learning curve analysis of NOSES. Graph of the raw operative time (OT) plotted against chronological case numbers for transvaginal specimen extraction **(A)** and transrectal specimen extraction **(B)** Cumulative sum (CUSUM) analyses based on OT for transvaginal **(C)** and transrectal **(D)** procedures are also presented. The red dashed line represents the best-fitted curve, and the green arrow indicates the inflection point of the learning curves. The best-fitted logarithmic model is shown in red.

Patients with right colon cancer who underwent NOSES performed by the same surgical team were included in this analysis. Specimen extraction through the vaginal and rectal routes was analyzed separately and is presented in Figure 1. Overall, operative time decreased significantly with accumulated experience, while postoperative recovery and complication rates remained stable across different phases, indicating a safe learning process.

The raw operative time (OT) for NOSES with transvaginal specimen extraction was plotted against the chronological case order (Figure 1A), showing a steady reduction. A similar trend was observed for transrectal specimen extraction (Figure 1B). These findings suggest a complex, nonlinear relationship between OT and surgical experience.

To further evaluate the learning curve, CUSUM analysis was applied using the mean OT as a reference point. As shown in Figure 1C, the CUSUM analysis for transvaginal specimen extraction was best modeled as a third-order polynomial. The curve exhibited a gradual upward slope until the 32nd case, followed by minor fluctuations between the 32nd and 57th cases, and a subsequent steep decline after the 57th case. Similarly, for transrectal specimen extraction (Figure 1D), OT showed a significant reduction after the 9th case, reaching a steady state after the 32nd case. Both transvaginal (Supplementary Table S1) and transrectal (Supplementary Table S2) NOSES were stratified into three sequential phases. Statistical analysis demonstrated a significant reduction in operative time across phases, reflecting improved technical efficiency. Importantly, no significant differences were observed among the three phases in terms of postoperative recovery parameters or postoperative complication rates (*P* > 0.05).

### 3.4 Perioperative fatigue levels

Perioperative fatigue outcomes are presented in Table 3. NOSES was associated with significantly lower early postoperative fatigue and a reduced incidence of postoperative fatigue syndrome during hospitalization. There was no significant difference in fatigue levels between the two groups one day before surgery (*P* = 0.792). However, postoperative fatigue score was significantly more reduced in the NOSES group. On postoperative day 1, the median fatigue score was 5 (4–6) in the NOSES group compared to 7 (6–8) in the TLRH group (*P* < 0.001). A similar trend was observed on postoperative day 3, with NOSES patients reporting lower fatigue scores [5 (3–6) vs. 6 (5–7), *P* < 0.001]. By postoperative day 7, fatigue levels had decreased and were comparable between the two groups (*P* = 0.589).

**Table 3:** Comparison of perioperative fatigue levels between TLRH and NOSES group.

Variable	TLRH	NOSES	*P*
	(*n* = 115)	(*n* = 115)	
Christensen score			
Preoperative 1 d	2 (1–3)	2 (1–3)	0.792
Postoperative 1d	7 (6–8)	5 (4–6)	**<0**.**001**
Postoperative 3 d	6 (5–7)	5 (3–6)	**<0**.**001**
Postoperative 7 d	4 (3–5)	4 (3–5)	0.589
Patients with POFS, *n* (%)			
Postoperative 1d	74 (64.3)	51 (44.3)	**0**.**002**
Postoperative 3 d	60 (52.2)	39 (33.9)	**0**.**005**
Postoperative 7 d	24 (18.3)	21 (18.3)	0.618

Values are presented as median and interquartile range, or numbers (%).

POFS, postoperative fatigue syndrome.

Bold values indicate *P* < 0.05.

Regarding POFS, defined as a Christensen score greater than 5, a significantly lower proportion of patients in the NOSES group experienced POFS on postoperative day 1 (44.3% vs.64.3%, *P* = 0.002) and day 3 (33.9% vs.52.2%, *P* = 0.005). By day 7, the incidence of POFS had declined in both groups, with no significant difference observed (18.3% in both, *P* = 0.618).

### 3.5 Postoperative functional outcomes

As summarized in Table 4, no significant differences were observed between the TLRH and NOSES groups in terms of pelvic floor function or fecal incontinence preoperatively.

**Table 4:** PFDI-20 and wexner scores between TLRH and NOSES group.

Variable	Preoperative Score		*P*	30-Day Postoperative Score		*P*	One-Year Postoperative Score		*P*
	TLRH	NOSES		TLRH	NOSES		TLRH	NOSES	
	(*n* = 115)	(*n* = 115)		(*n* = 115)	(*n* = 115)		(*n* = 115)	(*n* = 115)	
POPDI-6	3 (2–4)	3 (2–5)	0.225	3 (2–4)	3 (2–5)	0.175	3 (2–4)	3 (2–4)	0.145
CRADI-8	5 (4–6)	5 (4–6)	0.951	5 (4–6)	5 (4–6)	0.868	5 (4–6)	5 (4–6)	0.357
UDI-6	3 (2–4)	3 (2–4)	0.533	3 (2–4)	3 (2–4)	0.490	4 (2–4)	4 (2–4)	0.851
PFDI-20	11 (9–13)	11 (9–13)	0.782	11 (9–13)	11 (9–13)	0.752	12 (9–13)	12 (9–13)	0.659
Incontinence for flatus	1 (1–2)	1 (1–2)	0.598	2 (1–2)	1 (1–2)	0.599	2 (1–2)	1 (1–2)	0.547
Incontinence for liquid stools	2 (1–3)	2 (1–3)	0.142	2 (1–3)	2 (1–3)	0.197	2 (1–3)	2 (1–3)	0.147
Incontinence for solid stools	2 (1–3)	2 (1–3)	0.794	2 (1–3)	2 (1–3)	0.803	2 (2–3)	2 (1–3)	0.923
Wearing a pad	0 (0–1)	1 (0–1)	0.357	0 (0–1)	1 (0–1)	0.511	0 (0–1)	1 (0–1)	0.303
Lifestyle alteration	0 (0–0)	0 (0–0)	0.853	0 (0–0)	0 (0–0)	0.635	0 (0–0)	0 (0–0)	0.963
Wexner score	6 (5–7)	6 (5–7)	0.538	6(5–7)	6(5–7)	0.556	6(5–7)	6(5–7)	0.547

Values are presented as median and interquartile range.

POPDI-6, Pelvic Organ Prolapse Distress Inventory 6; CRADI-8, Colorectal-Anal Distress Inventory 8; UDI-6, Urinary Distress Inventory 6; PFDI-20, Pelvic Floor Distress Inventory Short Form 20.

Similarly, there were no significant differences in pelvic floor function between the TLRH and NOSES groups at 30 days, and 1 year postoperatively. Specifically, PFDI-20 scores and its subscales (POPDI-6, CRADI-8, and UDI-6) remained comparable between groups across all time points (all *P* > 0.05). A mild and transient increase in symptoms was observed at 30 days postoperatively, followed by recovery to baseline or near-baseline levels at 1 year. Importantly, no individual PFDI-20 domain-including POPDI-6, CRADI-8, or UDI-6-demonstrated a worsening trend in the NOSES group. Measures of fecal continence and Wexner scores also remained comparable between groups across all time points (all *P* > 0.05).

### 3.6 Survival

**Figure 2:**
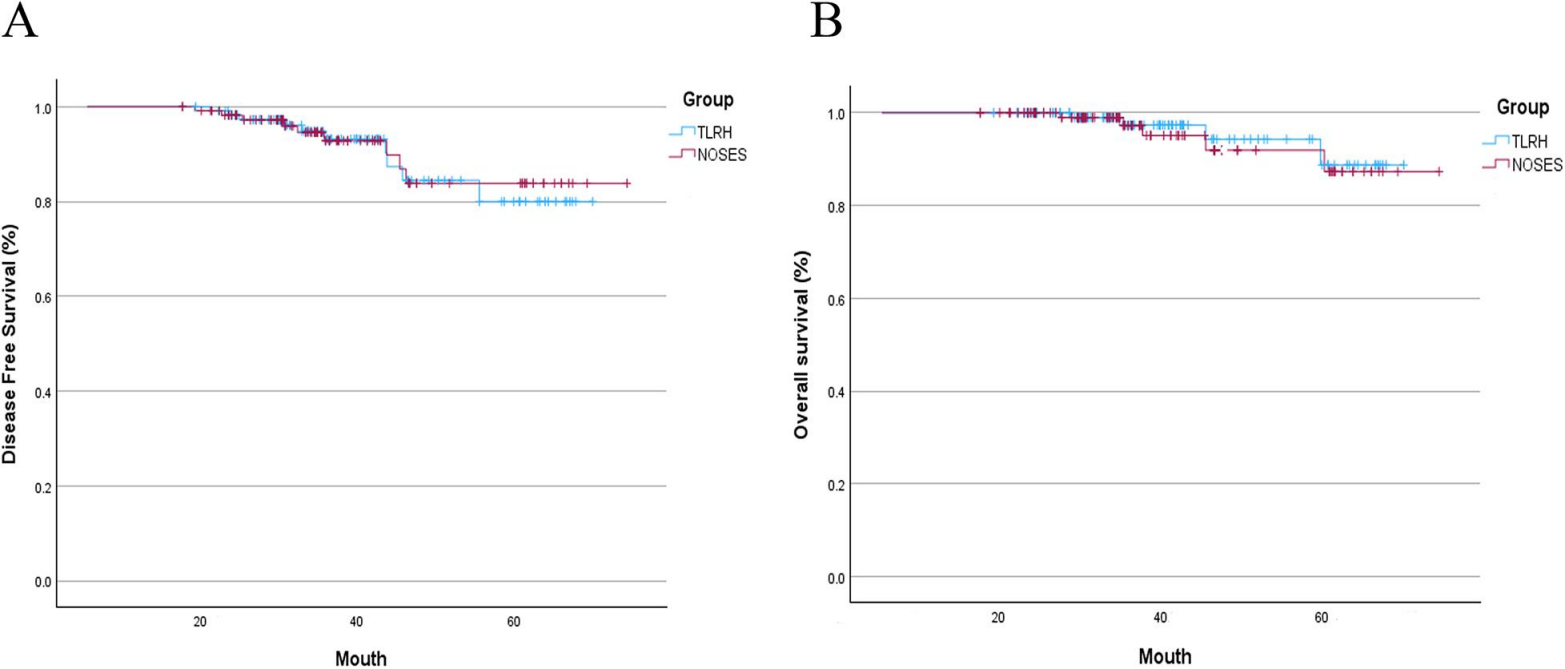
Kaplan–Meier analysis for comparison of disease-free survival (DFS) and overall survival (OS) of patients. **(A)** Comparison of DFS between the NOSES group and TLRH group. **(B)** Comparison of OS between NOSES and TLRH group.

Long-term oncologic outcomes demonstrated comparable disease control between the two groups. In a median follow-up time of 49 months (range 12–88 months) in TLRH group and 45.5 months (range 12–86 months) in NOSES group, no patients experienced local recurrence. Adjuvant therapy compliance and postoperative surveillance protocols were consistent across groups. However, distant metastasis developed in 9 patients from the NOSES group and 10 patients from the TLRH group. Additionally, 5 patients in the NOSES group and 4 patients in the TLRH group died due to metastatic disease. Despite these occurrences, there were no significant differences in DFS (Figure 2A) or OS (Figure 2B) between the two groups (*P* = 0.888 and *P* = 0.742, respectively).

## 4 Discussion and conclusions

This retrospective study evaluated 349 patients and, using PSM, compared the short- and long-term outcomes of 115 patients undergoing NOSES right hemicolectomy with those treated by TLRH. To our knowledge, this is the first PSM-based study directly comparing NOSES with intracorporeal anastomosis TLRH for right-sided colon cancer, building upon prior studies that primarily contrasted NOSES with conventional laparoscopic approaches. In addition to standard perioperative and oncologic outcomes, we incorporated rarely assessed endpoints, including postoperative fatigue, postoperative fatigue syndrome, functional recovery, and a quantitative learning curve analysis, providing new patient-centered and technical insights. We found that NOSES was associated with less postoperative pain, reduced analgesic use, fewer incision-related complications, and lower early postoperative fatigue, without adversely affecting overall complications, pelvic floor function, disease-free survival, or overall survival. These findings further support NOSES as a safe and effective minimally invasive option for right hemicolectomy.

A key finding of our study is that patients in the NOSES group experienced less postoperative pain and required fewer analgesics. This may be attributed to the lower sensitivity of visceral nerves compared to somatic nerves, resulting in milder pain perception and a more comfortable recovery (27, 28). Previous research has also demonstrated the analgesic benefits of NOSES. For instance, Li et al. (23) compared 32 patients who underwent transvaginal specimen extraction with those who had laparoscopic-assisted surgery and found significantly lower VAS scores on postoperative days 1, 3, and 5. Efetov and colleagues reported that combining reduced-port laparoscopic surgery with NOSES was associated with reduced postoperative pain on the first day (12). These results further establish NOSES as an effective approach for reducing postoperative pain, a key short-term advantage that can enhance recovery. Beyond pain control, minimizing postoperative discomfort plays a crucial role in reducing physiological stress responses, which, if triggered, may prolong recovery by increasing inflammation and delaying healing (29). Additionally, postoperative pain is closely linked to fatigue, sleep disturbances, and overall well-being (30), potentially contributing to postoperative fatigue syndrome (POFS). In our study, we observed that NOSES patients had lower fatigue scores on POD 1 and 3, with 44% vs. 64% exceeding the clinically relevant threshold on POD 1, representing a meaningful reduction in early postoperative fatigue. Lower fatigue in the early postoperative period is likely to facilitate earlier mobilization, greater participation in daily activities, and faster functional recovery, highlighting an under-recognized benefit of minimally invasive surgery (31). The reduced early fatigue in the NOSES group may be multifactorial, likely reflecting less postoperative pain, attenuated surgical stress and inflammatory response, consistent with prior observations that inflammation and pain contribute to postoperative fatigue (32). To our knowledge, this is the first report linking NOSES with reduced short-term postoperative fatigue and lower early POFS incidence during hospitalization. However, at three months postoperatively, no significant differences were observed in Christensen fatigue scores or POFS incidence, suggesting that the long-term impact of NOSES on fatigue remains uncertain.

We conducted a comprehensive analysis of the learning curve for NOSES, which has not been extensively explored in previous studies. This understanding is vital for the widespread adoption of NOSES, given its requirement for advanced laparoscopic skills and proficiency in intracorporeal anastomosis. We divided the learning process into three stages: initial, transition, and proficiency, based on operative time. CUSUM analysis showed that 32 cases were needed to begin the learning process for NOSES, with an additional 26 required to master transvaginal specimen extraction. In contrast, the learning curve for transrectal extraction was shorter, requiring 9 cases to reach the transition phase and 32 cases to achieve proficiency. This suggests that prior experience with transvaginal extraction may ease the adoption of transrectal techniques. Although research on the learning curve of NOSES is limited, studies on related procedures, such as intracorporeal intestinal anastomosis, provide valuable context. For instance, Torres et al. (33) found that 21 cases were sufficient to achieve competent anastomosis in laparoscopic surgery. More recent studies on laparoscopic right hemicolectomy with overlap anastomosis revealed that the learning curve flattened after just five cases for experienced surgeons (34). These findings suggest that intracorporeal anastomosis can be learned relatively quickly, while NOSES requires a longer learning period. This underscores the importance of surgical experience in improving postoperative outcomes. Therefore, structured training and hands-on practice are essential for successfully adopting NOSES and optimizing patient recovery.

A major concern with NOSES is its potential impact on pelvic and rectal function, as specimen extraction through the vagina or rectum may cause functional impairment. While previous studies have shown that transvaginal specimen extraction does not negatively affect postoperative sexual function (17), its broader impact on pelvic and rectal function remains a key consideration. To address this, our study evaluated these functions using the PFDI-20 and Wexner scores. In the present study, both surgical approaches demonstrated similar pelvic floor recovery trajectories, with no evidence of persistent or domain-specific impairment following NOSES. Although a mild short-term postoperative fluctuation was observed, symptoms resolved by 1 year in both groups. Our findings revealed no significant differences between the NOSES and TLRH, suggesting that NOSES does not compromise pelvic or rectal function. This consistency with prior research further reinforces the safety of NOSES regarding functional outcomes (23). Moreover, the absence of functional impairment may be attributed to meticulous intraoperative techniques, such as the use of muscle relaxants and gentle tissue handling, which help minimize potential risks.

The oncological safety of NOSES is still debated, but numerous studies and meta-analyses have found no significant differences between NOSES and laparoscopy regarding circumferential, distal, or proximal resection margins (35–37). Furthermore, research suggests that the NOSES group tends to have a greater lymph node yield, reinforcing its oncological safety (38). Our study echoed these results, showing no significant differences in resection margins or lymph node count between NOSES and TLRH. Long-term survival outcomes also support the safety of NOSES, with comparable 5-year OS and DFS rates observed between NOSES and TLRH. Our findings are consistent with larger multicenter studies, which reported that NOSES is oncologically safe when standard precautions are observed (7). We found similar results when comparing NOSES to TLRH, with no cases of local recurrence or vaginal/anal incision implantation during follow-up. While some studies have reported vaginal incision implantation due to the lack of an incision protector (39), this highlights the importance of using a specimen bag and protective sleeve to ensure oncological safety. Additionally, we found no difference in the incidence of distant metastases between the NOSES and TLRH groups, consistent with long-term research showing that transvaginal NOSES does not increase this risk (40). Together, these findings reinforce the growing body of evidence supporting the oncological safety of NOSES and its lack of impact on long-term outcomes.

In the matched cohort, operative time was slightly longer in the NOSES group than in the TLRH group (median 161 vs. 151 min), but this difference was not statistically significant (*P* = 0.191), with substantial overlap in interquartile ranges. Although previous studies and meta-analyses have reported a more pronounced increase in operative time for NOSES (14), several factors may explain the smaller difference observed in our series. All procedures were performed by an experienced colorectal surgical team in a high-volume center, and NOSES was introduced after technical proficiency in TLRH had already been achieved. Moreover, the standardized TLRH workflow in our center includes time-consuming steps such as intracorporeal mobilization, specimen preparation, and abdominal wall extraction and closure, particularly in patients with higher BMI, whereas NOSES avoids these steps. In addition, learning curve effects likely contributed: early NOSES cases required longer operative times, which were offset by more efficient performance in later cases.

This study has several limitations. First, the nonrandomized design may introduce selection bias and residual confounding despite the use of 1:1 propensity score matching. Although PSM minimized baseline differences between groups, unmeasured factors may still have influenced the outcomes. Second, the sample size and follow-up duration may not have been sufficient to detect subtle differences in long-term oncologic outcomes, warranting caution in interpreting survival results and underscoring the need for larger studies with extended follow-up. In addition, NOSES was applied in a carefully selected patient population according to current consensus recommendations, which may limit the generalizability of our findings. Future studies should evaluate whether the favorable perioperative and functional outcomes observed in this study can be extended to broader patient populations, including those with higher body mass index or larger tumors. Furthermore, surgical decision-making is influenced by patient preferences, tumor characteristics, and surgeon expertise. Future research should therefore incorporate patient-reported outcomes, such as health-related quality of life and cosmetic satisfaction, to better capture the full clinical value of NOSES. Multicenter randomized controlled trials with standardized techniques will be essential to further define the optimal role of NOSES in right-sided colon cancer surgery.

In conclusion, our study demonstrates that NOSES offers significant advantages over TLRH, including less postoperative pain, reduced fatigue, and fewer incision-related complications, while maintaining pelvic function and oncological outcomes. The learning curve analysis emphasizes the importance of surgical experience in refining the technique. These findings contribute to the growing evidence supporting NOSES as a safe and effective alternative to conventional laparoscopic surgery for right hemicolectomy. However, to strengthen these results and improve their generalizability, larger-scale, randomized controlled multicenter studies are needed.

## Data Availability

The raw data supporting the conclusions of this article will be made available by the authors, without undue reservation.

## References

[1] Bray F Laversanne M Sung H Ferlay J Siegel RL Soerjomataram I Global cancer statistics 2022: GLOBOCAN estimates of incidence and mortality worldwide for 36 cancers in 185 countries. CA Cancer J Clin. (2024) 74(3):229–63. 10.3322/caac.21834 38572751

[2] Buunen M Veldkamp R Hop WC Kuhry E Jeekel J Haglind E Survival after laparoscopic surgery versus open surgery for colon cancer: long-term outcome of a randomised clinical trial. Lancet Oncol. (2009) 10(1):44–52. 10.1016/S1470-2045(08)70310-3 19071061

[3] Stein SA Bergamaschi R. Extracorporeal versus intracorporeal ileocolic anastomosis. Tech Coloproctol. (2013) 17(Suppl 1):S35–9. 10.1007/s10151-012-0937-z 23250637

[4] Brown RF Cleary RK. Intracorporeal anastomosis versus extracorporeal anastomosis for minimally invasive colectomy. J Gastrointest Oncol. (2020) 11(3):500–7. 10.21037/jgo.2019.12.02 32655928 PMC7340812

[5] Hanna MH Hwang GS Phelan MJ Bui TL Carmichael JC Mills SD Laparoscopic right hemicolectomy: short- and long-term outcomes of intracorporeal versus extracorporeal anastomosis. Surg Endosc. (2016) 30(9):3933–42. 10.1007/s00464-015-4704-x 26715015

[6] Leung AL Cheung HY Li MK. Advances in laparoscopic colorectal surgery: a review on NOTES and transanal extraction of specimen. Asian J Endosc Surg. (2014) 7(1):11–6. 10.1111/ases.12070 24165166

[7] Guan X Hu X Jiang Z Wei Y Sun D Wu M Short-term and oncological outcomes of natural orifice specimen extraction surgery (NOSES) for colorectal cancer in China: a national database study of 5055 patients. Sci Bull (Beijing). (2022) 67(13):1331–4. 10.1016/j.scib.2022.05.014 36546264

[8] Zhang M Liu Z Wang X. Is natural orifice specimen extraction surgery the future direction of minimally invasive colorectal surgery? Surg Open Sci. (2022) 10:106–10. 10.1016/j.sopen.2022.08.001 36111268 PMC9467874

[9] Zhang ZC Luo QF Wang WS Chen JH Wang CY Ma D. Development and future perspectives of natural orifice specimen extraction surgery for gastric cancer. World J Gastrointest Surg. (2022) 14(11):1198–203. 10.4240/wjgs.v14.i11.1198 36504515 PMC9727573

[10] Efetov SK Tulina IA Kim VD Kitsenko Y Picciariello A Tsarkov PV. Natural orifice specimen extraction (NOSE) surgery with rectal eversion and total extra-abdominal resection. Tech Coloproctol. (2019) 23(9):899–902. 10.1007/s10151-019-02058-y 31482393

[11] Zhou Z Chen L Liu J Ji F Shang Y Yang X Laparoscopic natural orifice specimen extraction surgery versus conventional surgery in colorectal cancer: a meta-analysis of randomized controlled trials. Gastroenterol Res Pract. (2022) 2022:6661651. 10.1155/2022/6661651 35087585 PMC8789476

[12] Efetov SK Cao Y Panova PD Khlusov DI Shulutko AM. Reduced-port laparoscopic right colonic resection with D3 lymph node dissection and transvaginal specimen extraction (NOSES VIIIa) for right colon cancer: clinical features. Tech Coloproctol. (2024) 29(1):34. 10.1007/s10151-024-03055-6 39714748

[13] Huang X Wei R Li Q Qiu X Li P He W. Development and prospects of natural orifice specimen extraction surgery for colorectal cancer: a review article. Int J Surg. (2025) 111(4):2973–89. 10.1097/JS9.0000000000002285 39903566 PMC12175821

[14] Pompeu BF Mara Vieira Rocha V Machado Oliveira AF Marcolin P Dos Lucio Generoso LC Mazzola Poli De Figueiredo S Natural orifice specimen extraction for right-sided colon cancer: a systematic review and meta-analysis of propensity score-matched studies. Cureus. (2025) 17(5):e84191. 10.7759/cureus.84191 40376134 PMC12081068

[15] Zhang M Liu Z Sun P Hu X Zhou H Jiang Z Preliminary surgical outcomes of laparoscopic right hemicolectomy with transrectal specimen extraction: a propensity score matching study of 120 cases (with video). Gastroenterol Rep (Oxf). (2023) 11:goad036. 10.1093/gastro/goad036 37398927 PMC10313420

[16] Guan X Liu Z Longo A Cai JC Tzu-Liang Chen W Chen LC International consensus on natural orifice specimen extraction surgery (NOSES) for colorectal cancer. Gastroenterol Rep (Oxf). (2019) 7(1):24–31. 10.1093/gastro/goy055 30792863 PMC6375350

[17] Zhang M Hu X Guan X Zheng W Liu Z Jiang Z Surgical outcomes and sexual function after laparoscopic colon cancer surgery with transvaginal versus conventional specimen extraction: a retrospective propensity score matched cohort study. Int J Surg. (2022) 104:106787. 10.1016/j.ijsu.2022.106787 35922001

[18] Morton D Seymour M Magill L Handley K Glasbey J Glimelius B Preoperative chemotherapy for operable colon cancer: mature results of an international randomized controlled trial. J Clin Oncol. (2023) 41(8):1541–52. 10.1200/JCO.22.00046 36657089 PMC10022855

[19] Gosavi R Chia C Michael M Heriot AG Warrier SK Kong JC. Neoadjuvant chemotherapy in locally advanced colon cancer: a systematic review and meta-analysis. Int J Colorectal Dis. (2021) 36(10):2063–70. 10.1007/s00384-021-03945-3 33945007

[20] Fishman B Pasternak S Wallenstein SL Houde RW Holland JC Foley KM. The memorial pain assessment card. A valid instrument for the evaluation of cancer pain. Cancer. (1987) 60(5):1151–8. 10.1002/1097-0142(19870901)60:5<1151::AID-CNCR2820600538>3.0.CO;2-G 3300951

[21] Christensen T Bendix T Kehlet H. Fatigue and cardiorespiratory function following abdominal surgery. Br J Surg. (1982) 69(7):417–9. 10.1002/bjs.1800690721 7104617

[22] Nøstdahl T Bernklev T Fredheim OM Paddison JS Raeder J. Defining the cut-off point of clinically significant postoperative fatigue in three common fatigue scales. Qual Life Res. (2019) 28(4):991–1003. 10.1007/s11136-018-2068-0 30506178

[23] Asti E Bonavina L. Short-term efficacy of transvaginal specimen extraction for right colon cancer based on propensity score matching: a retrospective cohort study. Int J Surg. (2019) 70:28–9. 10.1016/j.ijsu.2019.08.008 31401324

[24] Ota E Nagasaki T Akiyoshi T Mukai T Hiyoshi Y Yamaguchi T Incidence and risk factors of bowel dysfunction after minimally invasive rectal cancer surgery and discrepancies between the wexner score and the low anterior resection syndrome (LARS) score. Surg Today. (2024) 54(7):763–70. 10.1007/s00595-023-02789-4 38170223

[25] Lin EL Sibona A Peng J Singh PN Wu E Michelotti MJ. Cumulative summation analysis of learning curve for robotic-assisted hiatal hernia repairs. Surg Endosc. (2022) 36(5):3442–50. 10.1007/s00464-021-08665-x 34327550

[26] Li XW Wang CY Zhang JJ Ge Z Lin XH Hu JH. Short-term efficacy of transvaginal specimen extraction for right colon cancer based on propensity score matching: a retrospective cohort study. Int J Surg. (2019) 72:102–8. 10.1016/j.ijsu.2019.07.025 31362128

[27] Zhu Z Wang KJ Orangio GR Han JY Lu B Zhou ZQ Clinical efficacy and quality of life after transrectal natural orifice specimen extraction for the treatment of middle and upper rectal cancer. J Gastrointest Oncol. (2020) 11(2):260–8. 10.21037/jgo.2020.03.05 32399267 PMC7212109

[28] Zhou ZQ Wang K Du T Gao W Zhu Z Jiang Q Transrectal natural orifice specimen extraction (NOSE) with oncological safety: a prospective and randomized trial. J Surg Res. (2020) 254:16–22. 10.1016/j.jss.2020.03.064 32402832

[29] Peters ML Sommer M de Rijke JM Kessels F Heineman E Patijn J Somatic and psychologic predictors of long-term unfavorable outcome after surgical intervention. Ann Surg. (2007) 245(3):487–94. 10.1097/01.sla.0000245495.79781.65 17435557 PMC1877005

[30] Lin X Feng X Sun L Wang Y Wu X Lu S Effects of esketamine on postoperative fatigue syndrome in patients after laparoscopic resection of gastric carcinoma: a randomized controlled trial. BMC Anesthesiol. (2024) 24(1):185. 10.1186/s12871-024-02513-w 38789968 PMC11127346

[31] Schwenk W Böhm B Müller JM. Postoperative pain and fatigue after laparoscopic or conventional colorectal resections. A prospective randomized trial. Surg Endosc. (1998) 12(9):1131–6. 10.1007/s004649900799 9716766

[32] Paddison JS Booth RJ Fuchs D Hill AG. Peritoneal inflammation and fatigue experiences following colorectal surgery: a pilot study. Psychoneuroendocrinology. (2008) 33(4):446–54. 10.1016/j.psyneuen.2007.12.011 18258374

[33] Torres Bermudez JR Buess G Waseda M Gacek I Becerra Garcia F Manukyan GA Laparoscopic intracorporal colorectal sutured anastomosis using the radius surgical system in a phantom model. Surg Endosc. (2009) 23(7):1624–32. 10.1007/s00464-008-9992-y 18553199

[34] Ozawa H Sakamoto J Nakanishi H Fujita S. Short-term outcomes of intracorporeal versus extracorporeal anastomosis after laparoscopic colectomy: a propensity score-matched cohort study from a single institution. Surg Today. (2022) 52(4):616–23. 10.1007/s00595-021-02375-6 34669014

[35] Zhou S Wang X Zhao C Pei W Zhou H Liu Q Comparison of short-term and survival outcomes for transanal natural orifice specimen extraction with conventional mini-laparotomy after laparoscopic anterior resection for colorectal cancer. Cancer Manag Res. (2019) 11:5939–48. 10.2147/CMAR.S209194 31303795 PMC6611704

[36] Hu JH Li XW Wang CY Zhang JJ Ge Z Li BH Short-term efficacy of natural orifice specimen extraction surgery for low rectal cancer. World J Clin Cases. (2019) 7(2):122–9. 10.12998/wjcc.v7.i2.122 30705889 PMC6354094

[37] Xingmao Z Haitao Z Jianwei L Huirong H Junjie H Zhixiang Z. Totally laparoscopic resection with natural orifice specimen extraction (NOSE) has more advantages comparing with laparoscopic-assisted resection for selected patients with sigmoid colon or rectal cancer. Int J Colorectal Dis. (2014) 29(9):1119–24. 10.1007/s00384-014-1950-7 24986143

[38] Xu S Liu K Chen X Yao H. The safety and efficacy of laparoscopic surgery versus laparoscopic NOSE for sigmoid and rectal cancer. Surg Endosc. (2022) 36(1):222–35. 10.1007/s00464-020-08260-6 33475847

[39] Gündoğan E Cicek E Sumer F Kayaalp C. A case of vaginal recurrence following laparoscopic left-sided colon cancer resection combined with transvaginal specimen extraction. J Minim Access Surg. (2019) 15(4):345–7. 10.4103/jmas.JMAS_182_18 30618419 PMC6839347

[40] Park JS Kang H Park SY Kim HJ Lee IT Choi GS. Long-term outcomes after natural orifice specimen extraction versus conventional laparoscopy-assisted surgery for rectal cancer: a matched case-control study. Ann Surg Treat Res. (2018) 94(1):26–35. 10.4174/astr.2018.94.1.26 29333423 PMC5765275

