# Supplementary material for: Comparative outcomes of natural orifice specimen extraction surgery versus totally laparoscopic surgery for right-sided colon cancer: a single-centre propensity score-matched study
Source: Front Surg. 2026 Feb 25;13:1716425. doi: 10.3389/fsurg.2026.1716425 (PMC12975889; doi:10.3389/fsurg.2026.1716425)
Supplement: Supplementary file 1 [file Table1.docx]

**Supplementary Table 1. Perioperative and postoperative outcomes across phases of transvaginal NOSES**

| **Variable** | **Phase I** | **Phase II** | **Phase III** | ***P*** |
| --- | --- | --- | --- | --- |
|  | (n=32) | (n=24) | (n=12) |  |
| Operative time, min | 170(150-205) | 162(138-190) | 145(125-170) | **<0.001** |
| Estimated blood loss, mL | 25(15-35) | 25(15-35) | 25(15-30) | 0.886 |
| 1st flatus, d | 2(2-3) | 2(2-3) | 2(2-3) | 0.963 |
| 1st defecation, d | 4(4-5) | 4(4-5) | 4(4-5) | 0.981 |
| Postoperative hospital stay, d | 6(4-7) | 6(5-7) | 6(5-7) | 0.902 |
| Postoperative complication, n (%) | 6(18.8) | 4(16.7) | 1(8.3) | 0.643 |
| Grade 1 | 5 | 2 | 1 |  |
| Grade 2 | 1 | 0 | 0 |  |
| Grade 3 | 0 | 1 | 0 |  |
| Grade 4 | 0 | 1 | 0 |  |
| Anastomotic fistula | 1 | 0 | 0 |  |
| Anastomotic bleeding | 1 | 1 | 0 |  |
| Intestinal obstruction | 1 | 0 | 0 |  |
| Abdominal infection | 1 | 0 | 0 |  |

Values are presented as median and interquartile range, or numbers (%).

**Supplementary Table 2. Interphase comparisons of peri/postoperative outcomes of transrectal NOSES**

| **Variable** | **Phase I** | **Phase II** | **Phase III** | ***P*** |
| --- | --- | --- | --- | --- |
|  | (n=9) | (n=22) | (n=16) |  |
| Operative time, min | 165(145-190) | 155(135-180) | 147(126-171) | **0.003** |
| Estimated blood loss, mL | 25(20-35) | 25(15-30) | 25(20-30) | 0.808 |
| 1st flatus, d | 2(2-3) | 2(2-3) | 2(2-3) | 0.977 |
| 1st defecation, d | 4(4-5) | 4(4-5) | 4(4-5) | 0.981 |
| Postoperative hospital stay, d | 6(5-7) | 6(5-7) | 6(5-7) | 0.936 |
| Postoperative complication, n (%) | 2(4.2) | 3(6.4) | 3(6.4) | 0.774 |
| Grade 1 | 1 | 2 | 2 |  |
| Grade 2 | 0 | 1 | 0 |  |
| Grade 3 | 1 | 0 | 1 |  |
| Grade 4 | 0 | 0 | 0 |  |
| Anastomotic fistula | 0 | 0 | 0 |  |
| Anastomotic bleeding | 1 | 0 | 0 |  |
| Intestinal obstruction | 0 | 1 | 0 |  |
| Abdominal infection | 0 | 0 | 1 |  |

Values are presented as median and interquartile range, or numbers (%).
